# Cell type specificity of glucocorticoid signaling in the adult mouse hippocampus

**DOI:** 10.1111/jne.13072

**Published:** 2021-12-22

**Authors:** Eva M. G. Viho, Jacobus C. Buurstede, Jari B. Berkhout, Ahmed Mahfouz, Onno C. Meijer

**Affiliations:** ^1^ Division of Endocrinology Department of Medicine Leiden University Medical Center Leiden The Netherlands; ^2^ Einthoven Laboratory for Experimental Vascular Medicine Leiden University Medical Center Leiden The Netherlands; ^3^ Department of Human Genetics Leiden University Medical Center Leiden The Netherlands; ^4^ Delft Bioinformatics Laboratory Delft University of Technology Delft The Netherlands; ^5^ Leiden Computational Biology Center Leiden University Medical Center Leiden The Netherlands

**Keywords:** corticosteroid receptors, hippocampus, single‐cell RNA sequencing, stress hormones, transcription regulation

## Abstract

Glucocorticoid stress hormones are powerful modulators of brain function and can affect mood and cognitive processes. The hippocampus is a prominent glucocorticoid target and expresses both the glucocorticoid receptor (GR: *Nr3c1*) and the mineralocorticoid receptor (MR: *Nr3c2*). These nuclear steroid receptors act as ligand‐dependent transcription factors. Transcriptional effects of glucocorticoids have often been deduced from bulk mRNA measurements or spatially informed individual gene expression. However, only sparse data exists allowing insights on glucocorticoid‐driven gene transcription at the cell type level. Here, we used publicly available single‐cell RNA sequencing data to assess the cell‐type specificity of GR and MR signaling in the adult mouse hippocampus. The data confirmed that *Nr3c1* and *Nr3c2* expression differs across neuronal and non‐neuronal cell populations. We analyzed co‐expression with sex hormones receptors, transcriptional coregulators, and receptors for neurotransmitters and neuropeptides. Our results provide insights in the cellular basis of previous bulk mRNA results and allow the formulation of more defined hypotheses on the effects of glucocorticoids on hippocampal function.

## 1 INTRODUCTION

In the brain, stress responses and memory formation are essential to cope with changes in the environment.^1^ The hippocampus is crucial in these processes, and highly sensitive to fluctuating levels of glucocorticoid (GC) stress hormones.^2^, ^3^ GC levels naturally vary along the day following circadian and ultradian rhythms,^4^ and basal levels of endogenous GCs in the hippocampus are necessary for neuronal integrity, growth, differentiation and synaptic plasticity.^5^ Although acute stress induces only a temporary deviation from this balance, chronic stress or excessive GC exposure can threat the hippocampal homeostasis. All of these effects are mediated by the two types of corticosteroid receptors that are expressed in the brain: the glucocorticoid receptor (GR) and the mineralocorticoid receptor (MR). GR and MR are nuclear steroid receptors that can act as ligand‐dependent transcription factors (TFs). MR has a high GC affinity (*K*_d_ ~ 0.5 nm) and accordingly is activated substantially at basal hormone levels. GR has a lower affinity (*K*_d_ ~ 5 nm) and is therefore responsive to circadian GC peaks and fluctuations in the stress range.^6^ Binding studies, immunohistochemistry and *in situ* hybridization showed that expression of the *Nr3c2* gene (coding for MR) is mainly restricted to the limbic brain, specifically the hippocampus, whereas the *Nr3c1* gene (coding for GR) is widely expressed throughout the brain.^7^

To date, all genome‐wide studies on GR‐ and MR‐mediated transcription in the hippocampus have been conducted with bulk tissue mRNA measurements. However, the hippocampus is a complex brain structure with a wide diversity of neuronal as well as non‐neuronal cells, and with a particular spatial organization. Single‐cell RNA sequencing (scRNA‐seq) has allowed for a large‐scale comprehensive molecular classification of cell types in the brain.^8^, ^9^, ^10^ The Allen Institute for Brain Science recently sequenced approximately 1.2 million cells covering all regions of the adult mouse isocortex and hippocampal formation, identifying almost 380 subtypes of cells. The hippocampal data includes information on glutamatergic neurons from the dentate gyrus (DG) and cornu ammonis regions, GABAergic neurons, astrocytes, oligodendrocytes, microglia and endothelial cells.^11^ Our previous *in situ* hybridization‐based analysis on whole brain revealed spatially specific co‐expression patterns of *Nr3c1* and *Nr3c2* with genes that are responsive to GCs or involved in nuclear receptor transcriptional regulation. This suggested mechanisms for regional and cellular functional specificity of GC signaling.^12^ The advances in scRNA‐seq carry with them new computational methods to address such co‐expression at the cell type level, and allow the reconstruction of TF downstream pathways.^13^, ^14^, ^15^

In the present study, we used existing scRNA‐seq data^11^ to molecularly characterize the cellular heterogeneity of GR and MR signaling in the adult mouse hippocampus. We assessed cell type expression specificity of GR and MR downstream target genes to identify putative markers for GC responsiveness in particular cell types. Furthermore, we looked into GR and MR co‐expression with sex hormone receptors, transcriptional coregulators, and receptors for neurotransmitters and neuropeptides to define for each cell type the potential pathways that may interact with hippocampal GC signaling.

## 2 MATERIALS AND METHODS

### 2.1 scRNA‐seq data resources

The present study is based on the 10x scRNA‐seq dataset published by the Allen Institute for Brain Science^11^ (https://portal.brain‐map.org/atlases‐and‐data/RNA‐seq/mouse‐whole‐cortex‐and‐hippocampus‐10x). Briefly, the single cells were isolated from 16 different regions of the isocortex and the hippocampal formation from 54 male and female mice. The Allen Mouse Brain Common Coordinate Framework version 3 (CCFv3) ontology was used to define brain regions for profiling and boundaries for dissections. scRNA‐seq data from the regions of interest were generated using 10x Genomics Chromium. For downstream processing, cells with <1500 detected genes as well as doublets were filtered out. The data was then clustered, and cluster names were assigned based on the Allen Institute proposal for cell type nomenclature (https://portal.brain‐map.org/explore/classes/nomenclature). The topology of the taxonomy allowed to define the sex of the mouse from which the cells were isolated, the regions of interest, cell classes (glutamatergic, GABAergic or non‐neuronal) and subclasses.^11^, ^16^ This information was stored in the metadata table.

### 2.2 scRNA‐seq data metrics and pre‐processing

**FIGURE 1:**
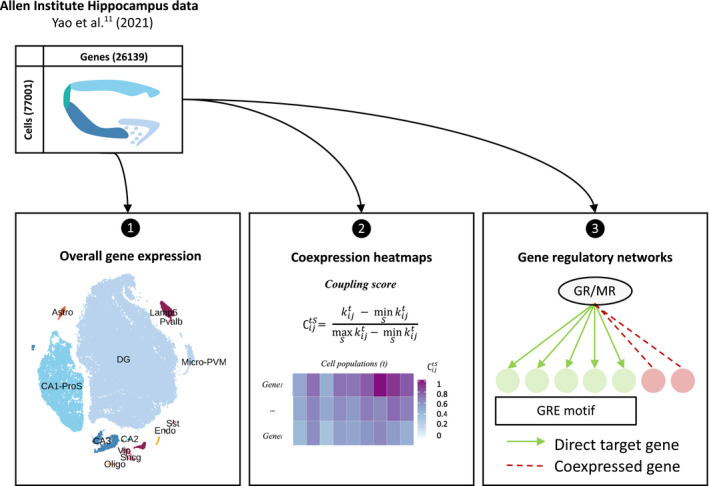
Schematic overview of the research strategy. Abbreviations: Astro, astrocytes; Oligo, oligodendrocytes; Endo, endothelial cells; micro‐PVM, microglia/perivascular macrophages; Lamp5, lysosomal associated membrane protein family member 5; Vip, vasoactive intestinal peptide; Pvalb, parvalbumin; Sncg, synuclein gamma; Sst, somatostatin; DG, dentate gyrus; CA1‐ProS, cornus ammonis 1‐prosubiculum; CA2, cornus ammonis 2; CA3, cornus ammonis 3; ${∁}_{\mathrm{ij}}^{\mathrm{tS}}$, coupling score, GR, glucocorticoid receptor; MR, mineralocorticoid receptor; GRE, glucocorticoid response element

The metadata was used to subset cells of the hippocampus region from the gene expression matrix. We selected for 13 subclasses of hippocampal cells. The final gene count matrix consisted of 77,001 cells for 26,139 genes (Figure 1) and was pre‐processed in R, version 3.6.1 (R Foundation for Statistical Computing) according to the Seurat, version 3.1.5 (https://satijalab.org/seurat) pipeline for quality control, normalization and analysis of scRNA‐seq data using the following criteria: *min*.*cells* = *1*, *min*.*features* = *100*, *normalized*.*method* = *LogNormalize*, *scale*.*factor* = *10000*,* selection*.*method* = *“vst”*,* nfeatures* =*2000*. The gene counts were normalized and log‐transformed across all cells, which allowed for statistical comparison between cells and cell types, as described previously.^17^ We performed principal component analysis and we selected the top 50 PCs as input for the *t*‐distributed stochastic neighbor embedding (t‐SNE) dimensional reduction. Finally, the transcriptomic data was analyzed and displayed using Seurat visualization tools (Figure 1).

### 2.3 Bulk RNA sequencing of mouse ventral hippocampus

The animal study was approved by the ethics committee of local Animal Committee of the University of Amsterdam. Eight‐week‐old C57BL/6 J male mice were group‐housed by four in conventional cages under a 12:12 hour light/dark photocycle and had access to food and water available ad libitum. Mice received an injection of either 3 mg.kg^–1^ corticosterone (*n* = 4) or vehicle (*n* = 4) between 9:00 and 10:00 a.m. Mice were killed by decapitation 3 h after injection. The ventral hippocampus was collected for mRNA sequencing (RNA‐seq). Total RNA was isolated with the NucleoSpin^®^ RNA kit (Macherey‐Nagel) and RNA quality was assessed using the RNA 6000 Nano kit on Bioanalyzer (Agilent). All samples had an RNA Integrity Number over 6.5 with a 28/18s ratio over 1, and therefore were considered suitable for sequencing. Aliquots of total RNA samples were sent for transcriptome sequencing at BGI Genomics. Stranded mRNA libraries were constructed and 100‐bp paired end sequencing was performed on the DNBseq platform, resulting in over 20 million reads per sample. RNA‐seq data have been deposited in the NCBI Gene Expression Omnibus and are accessible through GEO Series accession number GSE184924. The Gentrap pipeline, published as part of Bio Pipeline Execution Toolkit (Biopet, https://biopet‐docs.readthedocs.io), was used for reads quality control, alignment and quantification. Quality control was performed using FastQC and MultiQC. Reads were aligned 10 mm using GSnap aligner, version 2017‐09‐11. The gene‐read quantification was performed using HTSeq‐count, version 0.6.0. HTSeq‐count output files were merged into a count matrix as input for differential gene expression analysis. DEseq2, version 1.29.4,^18^ was used for normalization of the data (median of ratio’s method) and identification of differentially expressed genes in R, version 3.4. The differential expression analysis, resulting in 16,839 genes in the analysis. The contrast between vehicle and corticosterone groups was analyzed for differential expression in a pairwise comparison. An false discovery rate adjusted *p* value of .05 was used as a cut‐off to determine differentially expressed genes.

### 2.4 Selection of gene sets

*Steroid receptors*: This gene set contains the stress and sex hormones nuclear steroid receptors, the GR (*Nr3c1* – nuclear receptor subfamily 3 group C member 1), the MR (*Nr3c2* – nuclear receptor subfamily 3 group C member 2), the androgen receptor (*Ar*), the progesterone receptor (*Pgr*), and the estrogen receptors *α* and *β* (*Esr1* and *Esr2*).
*GR and MR target genes*: This set of genes is based on previous transcriptomic studies in rodent brain and neuronal cells after glucocorticoid treatment,^19^ our recent RNA‐seq results in mouse ventral hippocampus after corticosterone injection, and two chromatin immunoprecipitation followed by sequencing (ChIP‐seq) studies on GR and MR after injection with either 0.3 or 3 mg.kg^–1^ corticosterone in rats.^20^, ^21^ The criteria for ‘target genes’ were (1) regulation by GCs in previously published studies on rodent brain and (2) in our recent transcriptomic results, given that these exclusively represent mouse hippocampus; (3) the direction of regulation had to be consistent in all reporting studies; and (4) the gene had to be associated with a binding site for either GR, MR or both receptors according to the two ChIP‐seq studies that we used. The latter were in rat hippocampus, but it has become apparent that functional GC response elements (GREs) tend to be evolutionary conserved.^22^, ^23^
*Coregulators*: The gene set of GR and MR AF‐2 coregulators was based on previous profiling analysis published by Broekema et al.^24^
*Neurotransmitter and neuropeptides receptor repertoire*: We aimed for an exhaustive list of genes for the adrenergic, serotoninergic, cholinergic and dopaminergic receptors according to the HUGO Gene Nomenclature Committee at the European Bioinformatics Institute (HGNC database: https://www.genenames.org). The neuropeptides receptors list was based on the HGNC database and the previous study from Smith et al.^25^ on intracortical neuropeptide networks.

### 2.5 scRNA‐seq coupling matrices for *Nr3c1* and *Nr3c2* co‐expression profiles

A coupling score of *Nr3c1* and *Nr3c2* with genes of interest was calculated to rank their co‐expression. First, we calculated the average expression of each gene of interest *i* in cell type *t* ($x_{i}^{t}$), where *t* is one of the 13 cell types in the adult mouse hippocampus. For each corticosteroid receptor (*Nr3c1* and *Nr3c2*), we calculated the coupling score as previously described,^25^ as $k_{\mathrm{ij}}^{t}=\;\log_{10}\left(x_{i}^{t}\;\times \;x_{j}^{t}\right)$, where i∈S and S is one of the gene sets described earlier, and j∈{Nr3c1,Nr3c2}. For each gene set S, we calculated the normalized coupling score ${∁}_{\mathrm{ij}}^{\mathrm{tS}}$ (Figure 1):

$${∁}_{\mathrm{ij}}^{\mathrm{tS}}=\;\frac{k_{\mathrm{ij}}^{t}\;‐\;\min_{S}k_{\mathrm{ij}}^{t}}{\max_{S}k_{\mathrm{ij}}^{t}‐\;\min_{S}k_{\mathrm{ij}}^{t}}$$

### 2.6 pySCENIC: Assessment of GR and MR single cell gene regulatory network activity

The gene expression matrix of the clustered hippocampus scRNA‐seq dataset underwent the scalable Python SCENIC (pySCENIC) (https://pyscenic.readthedocs.io) workflow for single‐cell gene regulatory network (GRN) analysis as described by Van de Sande et al.^15^ pySCENIC reconstructs GRNs (i.e., TFs together with their target genes) and assesses the *de novo* GRN activity in individual cells (Figure 1). The pySCENIC workflow, version 0.10.3, was performed under Python, version 3.8.5 (https://www.python.org) and the output was then processed with Seurat, version 3.1.5 in R, version 3.6.1.

### 2.7 Differential expression and GRN activity analysis of scRNA‐seq data

The gene count matrix for hippocampal gene expression and the GRN activity matrix underwent differential expression/activity analysis to identify genes specifically more expressed or GRNs specifically more active in certain cell types. Both differential analyses were performed using the Seurat FindAllMarkers function (Wilcoxon rank sum test)^17^ in R, version 3.6.1. Furthermore, significant differences in gene expression throughout cell types or within one cell type were tested with a paired two‐sided Wilcoxon test (wilcox.test function) on average expression in R, version 3.6.1.

### 2.8 Code availability

Open‐source algorithms were used as described for single‐cell analysis methods^17^ and GRNs analysis.^15^ Details on how these algorithms were used, as well as the code for coupling score calculation, are available in the GitHub repository (https://github.com/eviho/10XHip2021_VihoEMG).

## 3 RESULTS

### 3.1 *Nr3c1* (GR) and *Nr3c2* (MR) expression show significant cell specificity across hippocampal cell types

**FIGURE 2:**
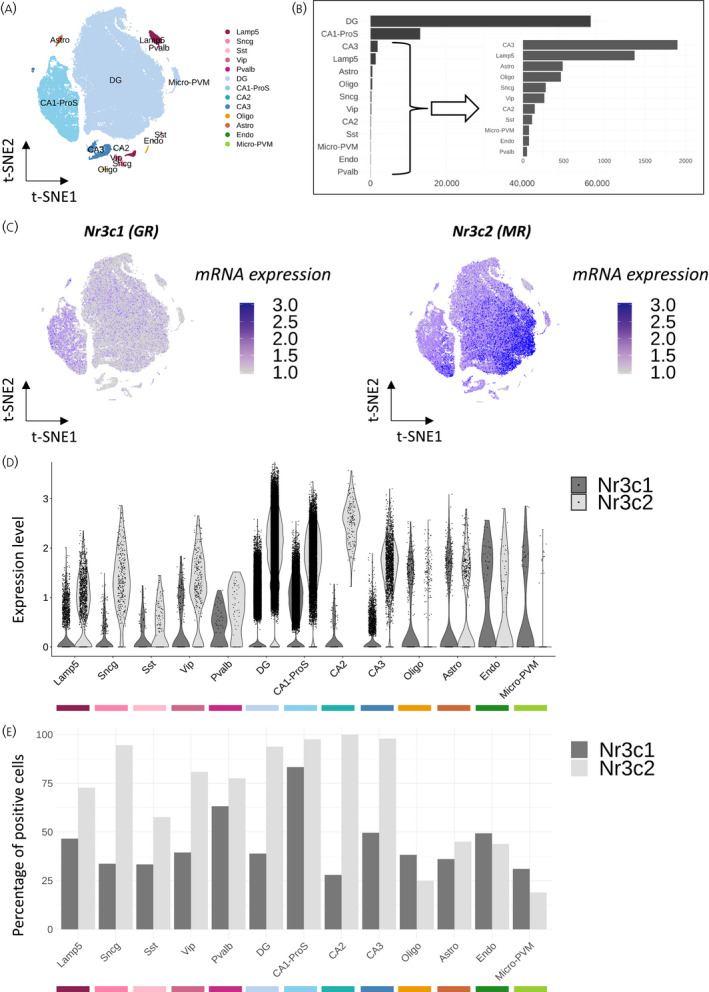
Cell type specificity of *Nr3c1* and *Nr3c2* expression in the adult mouse hippocampus. (A) Dimensional reduction (t‐SNE) representation of mouse hippocampal cells grouped by gene expression profile similarities and assigned to known cell types. (B) Number of cells per cell type within the dataset. (C) t‐SNE representation of *Nr3c1* and *Nr3c2* log‐normalized mRNA expression per cell, scaled from 1 to 3 (mRNA expression). (D) Violin plot of *Nr3c1* and *Nr3c2* log‐normalized expression (Expression level). (E) Bar plot of the percentage of cells positive for *Nr3c1* and *Nr3c2*. Abbreviations: t‐SNE, t‐distributed stochastic neighbor embedding; Nr3c1, nuclear receptor subfamily 3 group C member 1; Nr3c2, nuclear receptor subfamily 3 group C member 2; GR, glucocorticoid receptor; MR, mineralocorticoid receptor; Astro, astrocytes; Oligo, oligodendrocytes; Endo, endothelial cells; Micro‐PVM, microglia/perivascular macrophages; Lamp5, lysosomal associated membrane protein family member 5; Vip, vasoactive intestinal peptide; Pvalb, parvalbumin; Sncg, synuclein gamma; Sst, somatostatin; DG, dentate gyrus; CA1‐ProS, cornus ammonis 1‐prosubiculum; CA2, cornus ammonis 2; CA3, cornus ammonis 3

Our approach aimed to describe the diversity of corticosteroid receptors *Nr3c1* (GR) and *Nr3c2* (MR) signaling networks in mouse hippocampal cell types, using publicly available scRNA‐seq data. We selected hippocampal cells from the Yao et al.^11^ mouse brain dataset, which resulted in 77,001 cells, divided over 13 different cell types (Figure 2A). The most abundant cell types in this dataset were the DG and cornu ammonis 1/pro‐subiculum (CA1‐ProS) glutamatergic neurons with 58,566 and 13,221 cells, respectively. The two last glutamatergic neuron populations CA2 and CA3 contained 143 and 1899 cells, respectively. GABAergic neurons were divided into five subtypes containing between 49 and 1372 cells: parvalbumin (Pvalb), somatostatin (Sst), vasoactive intestinal peptide (Vip), synuclein gamma (Sncg) and lysosomal associated membrane protein family member 5 (Lamp5) positive neurons. Finally, the data revealed four non‐neuronal cell types: 488 astrocytes (Astro), 465 oligodendrocytes (Oligo), 73 endothelial cells (Endo) and 74 microglial cells/perivascular macrophages (micro‐PVM) (Figure 2B).

We assessed *Nr3c1* and *Nr3c2* relative expression levels throughout the hippocampal cell types. Although the t‐SNE representation clearly showed a significant higher expression level of *Nr3c2* compared to *Nr3c1* in the mouse hippocampus (log_2_FC = 2.82, *p* = .02) (Figure 2C), the data was biased towards the most abundant cell types (DG and CA1‐ProS). Per population, we observed a relatively higher expression of *Nr3c2* compared to *Nr3c1* in glutamatergic neurons, which was more pronounced in CA2 (log_2_FC = 3.74, *p* < .001) (Figure 2D). *Nr3c2* was actually enriched in CA2 (log_2_FC = 0.53, *p* < .001) and the DG (log_2_FC = 0.32, *p* < .001) compared to other cell types (see Table S1). Interestingly, *Nr3c2* was also more expressed than *Nr3c1* in GABAergic neurons with the biggest difference in Sncg neurons (log_2_FC = 2.75, *p* < .001) (see Table S1). *Nr3c1* was more expressed in non‐neuronal cell types with the biggest contrast in micro‐PVM cells where *Nr3c2* was almost absent (Figure 2D). These differences in expression levels were in line with the percentage of cells expressing *Nr3c1* and *Nr3c2*. Between 50% and 100% of neurons (glutamatergic and GABAergic) were positive for *Nr3c2*, whereas only CA1‐ProS, CA3 and Pvalb types passed the 50% threshold of positive cells for *Nr3c1*. Regarding non‐neuronal types, they contained <50% cells positive for either *Nr3c1* or *Nr3c2*, with a slightly higher percentage of positive cells for *Nr3c1* compared to *Nr3c2* in oligodendrocytes, microglial and endothelial cells (Figure 2E).

Altogether, the results suggest a relatively higher basal expression of *Nr3c2* in mouse hippocampal neurons and astrocytes, whereas *Nr3c1* is relatively more expressed in oligodendrocytes, microglia and endothelial cells.

### 3.2 Classic GR and MR target genes differentially express across hippocampal cell types

**FIGURE 3:**
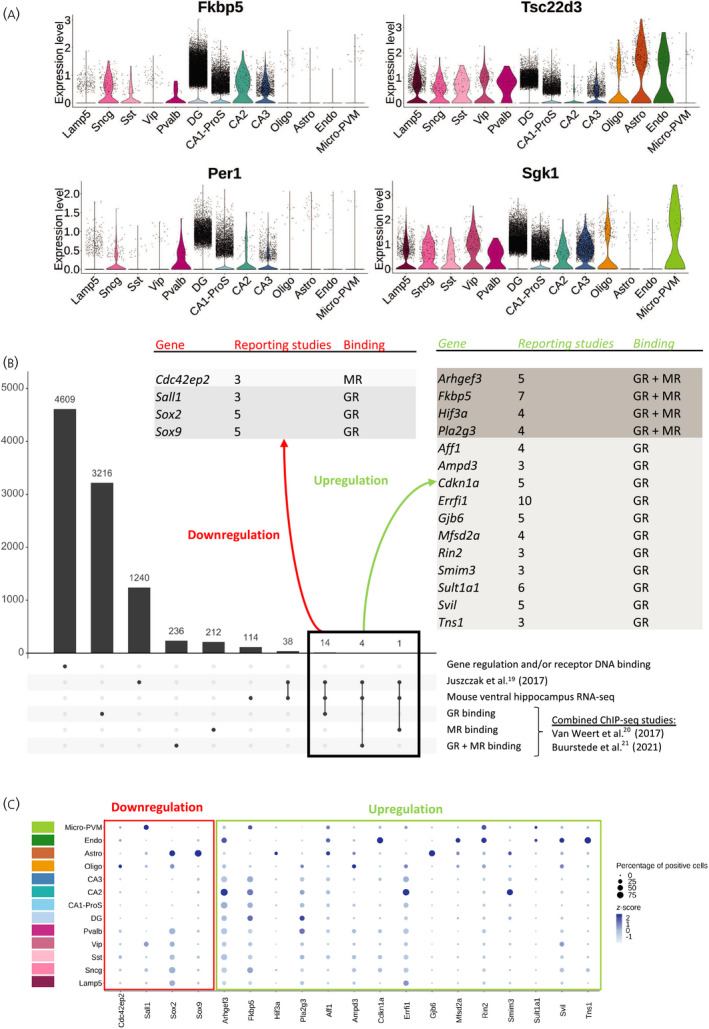
Cell type specificity of glucocorticoid target genes in the adult mouse hippocampus. (A) Violin plots representing the log‐normalized expression of commonly measured glucocorticoid responsive genes *Fkbp5*, *Tsc22d3*, *Per1* and *Sgk1* (Expression level). (B) List of new GR and MR target genes selection based on transcriptomic and DNA binding studies, associated with the number of transcriptomic studies reporting the gene (*reporting studies*), and DNA binding by GR, MR or both receptors (*binding*). (C) Dot plot representing both the centered log‐normalized average expression (*z*‐score) and the percentage of positive cells for the genes newly identified as GR and MR targets. Abbreviations: GR, glucocorticoid receptor; MR, mineralocorticoid receptor; ChIP, chromatin immunoprecipitation; RNA‐seq, RNA sequencing; Astro, astrocytes; Oligo, oligodendrocytes; Endo, endothelial cells; micro‐PVM, microglia/perivascular macrophages; Lamp5, lsosomal associated membrane protein family member 5; Vip, vasoactive intestinal peptide; Pvalb, parvalbumin; Sncg, synuclein gamma; Sst, somatostatin; DG, dentate gyrus; CA1‐ProS, cornus ammonis 1‐prosubiculum; CA2, cornus ammonis 2; CA3, cornus ammonis 3

Transcription‐dependent GC responsiveness of the hippocampus relies by definition on the presence of various GR and MR target genes. We investigated the basal expression of GC regulated genes in different hippocampal cell types. A limited class of genes is commonly measured in bulk brain mRNA to assess GC effects.^26^, ^27^, ^28^, ^29^, ^30^ This set includes FK506‐binding protein 5 (*Fkbp5*), glucocorticoid‐induced leucine zipper protein (*Tsc22d3*), period circadian regulator 1 (*Per1*) and serum/glucocorticoid regulated kinase 1 (*Sgk1*). However, the scRNA‐seq data showed a clear heterogeneity for the basal expression of these genes in different hippocampal cell types (Figure 3A). *Fkbp5* expression was predominant in glutamatergic neurons, particularly in the DG. In comparison, *Tsc22d3* was more expressed in GABAergic neurons and non‐neuronal cells than *Fkbp5*. Furthermore, the basal expression of *Per1* suggested high cell specificity, with high expression in only five neuronal cell types. Finally, *Sgk1* was expressed in most hippocampal cell types, but was absent in astrocytes and endothelial cells (Figure 3A). The average expression was in line with the percentage of cells expressing the genes of interest. On average, 50% of glutamatergic neurons expressed *Fkbp5*, whereas 50% of GABAergic neurons expressed *Tsc22d3*. *Sgk1* was more present in oligodendrocytes and microglia, whereas *Tsc22d3* was more present in astrocytes and endothelial cells (see Figure S1A). *Per1* was generally less expressed than any other classic target genes in the whole hippocampus, which might be partially explained by circadian variation (Figure 3A; see also Figure S1A). Although the analysis is performed on hippocampal basal gene expression, the results suggest an heterogenous and cell type‐specific response to GC signaling activation.

Regarding MR‐specific target genes, MR binding to DNA on GREs was described to be associated with NeuroD factor binding^31^ and *Jdp2* was found as an MR target gene in conjunction with MR/NeuroD binding. At the basal level in the scRNA‐seq data, *Neurod2* was mostly expressed in glutamatergic neurons and, although relatively few cells were positive for *Jdp2*, those expressing it were also glutamatergic neurons (see Figure S1B). *Nr3c2* expression in the DG differed throughout the cell population (Figure 2C). Therefore, we assessed DG cells using a deeper level of clustering. DG cells could be further divided into six distinct subclusters.^11^, ^16^ The most abundant cluster was 125_DG, where both *Nr3c2* and *Neurod2* still showed different levels of expression across the cell cluster, with a similar overall pattern (see Figure S1C). This suggests that, despite differentially expressing *Nr3c2* and *Neurod2*, cells in cluster 125_DG were not sufficiently divergent in the rest of their gene expression profile to be subdivided into more cell clusters. *Jdp2* was mainly expressed in cluster 122_DG and 125_DG. However, in the absence of GC treatment, *Jdp2* expression did not strongly correlate with the contrasted expression of *Nr3c2* or *Neurod2* in the DG (see Figure S1C).

### 3.3 A wider set of GC target genes further reveals GR and MR signaling heterogeneity across cell types

Although classic GC responsive genes already showed cellular heterogeneity of gene expression, we expanded the list of GC responsive genes to give a better recapitulation of cellular specificity of GR and MR signaling in the mouse hippocampus. We combined a published meta‐analysis on GC responsive genes in rodent and human brain (17 studies)^19^ with a recent RNA‐seq dataset that we obtained in mouse ventral hippocampus, as well as ChIP‐seq data assessing GR and MR DNA binding in rat hippocampus^20^, ^21^(see Table S2). This resulted in a list of 4609 genes either responsive to GC treatment or associated with a receptor binding site. Among those genes, 3216 reported GR‐specific binding to the DNA, 212 MR‐specific binding, and 236 reported both GR and MR binding. A total of 1240 genes were reported to be regulated in the previously published meta‐analysis, and 114 genes were GC responsive in our recent mouse hippocampus RNA‐seq dataset. We first selected for genes that were reported consistently in between the previously published meta‐analysis^19^ and our transcriptomic analysis. This subset of 38 genes was further filtered for genes that reported DNA binding of either GR, MR or both receptors in the ChIP‐seq studies. In total, 19 genes survived all criteria and were reported in at least three transcriptomic studies. Of these, four genes were consistently downregulated and 15 were consistently upregulated. *Cdc42ep2* was the only gene associated with MR binding, and a total of 14 genes were associated with exclusive GR binding and four genes were associated with both GR and MR binding, including *Fkbp5* (Figure 3B). *Tsc22d3*, *Per1* and *Sgk1* were previously reported in both transcriptomic and ChIP‐seq studies but absent in the recent mouse hippocampus RNA‐seq dataset (see Table S2).

The new subset of GR and MR target genes was further analyzed in the hippocampus scRNA‐seq data. Similar to the classic GC responsive genes, the new targets displayed a large heterogeneity in cell type basal expression (Figure 3C). Genes known to be downregulated after GC treatment showed high specificity for non‐neuronal cell types. *Cdc42ep2* was relatively more expressed in oligodendrocytes, *Sall1* in microglia, and *Sox2* and *Sox9* in astrocytes. Among genes known to be upregulated after GC treatment, more than half were relatively more expressed in non‐neuronal cells in these basal conditions. However, *Fkbp5* and *Pla2g3* were predominantly neuron specific. Moreover, *Arhgef3*, *Errfi1* and *Smim3* were preferentially expressed in CA2 (Figure 3C). We also investigated the cell type specificity of genes known to be regulated by GCs but not associated with a receptor binding site. In this list of 19 genes, three were not detectable in the scRNA‐seq data (*1810011O10Rik*, *Rhou*, *Lcn2*). Many genes were highly expressed in astrocytes (e.g., *Dio2*), two downregulated genes (*Abi3*, *Ccr5*) were microglia specific, and three genes were widely expressed in neurons but at low levels, except for *Ccng1* which was highly expressed and abundant in CA1‐ProS (see Figure S1D).

The results for GR and MR downstream target genes again highlighted the expression heterogeneity of GC target genes in mouse hippocampal cell types. Furthermore, under basal conditions, many target genes were specifically expressed in non‐neuronal cells. This indicates that transcripts from non‐neuronal cells may represent a substantial part of GC target genes.

### 3.4 *Nr3c1* and *Nr3c2* co‐expression with sex hormone receptors suggests cell type‐specific crosstalk

Corticosteroid receptors belong to the nuclear receptor superfamily that also includes the sex hormone receptors: the progesterone receptor (PR, coded by *Pgr*), androgen receptor (AR, coded by *Ar*), and estrogen receptors α and β (ERα and ERβ, coded by *Esr1* and *Esr2*). Sex steroid receptors may interact with MR and GR, but direct interactions would by definition depend on presence and co‐expression.^32^, ^33^, ^34^

**FIGURE 4:**
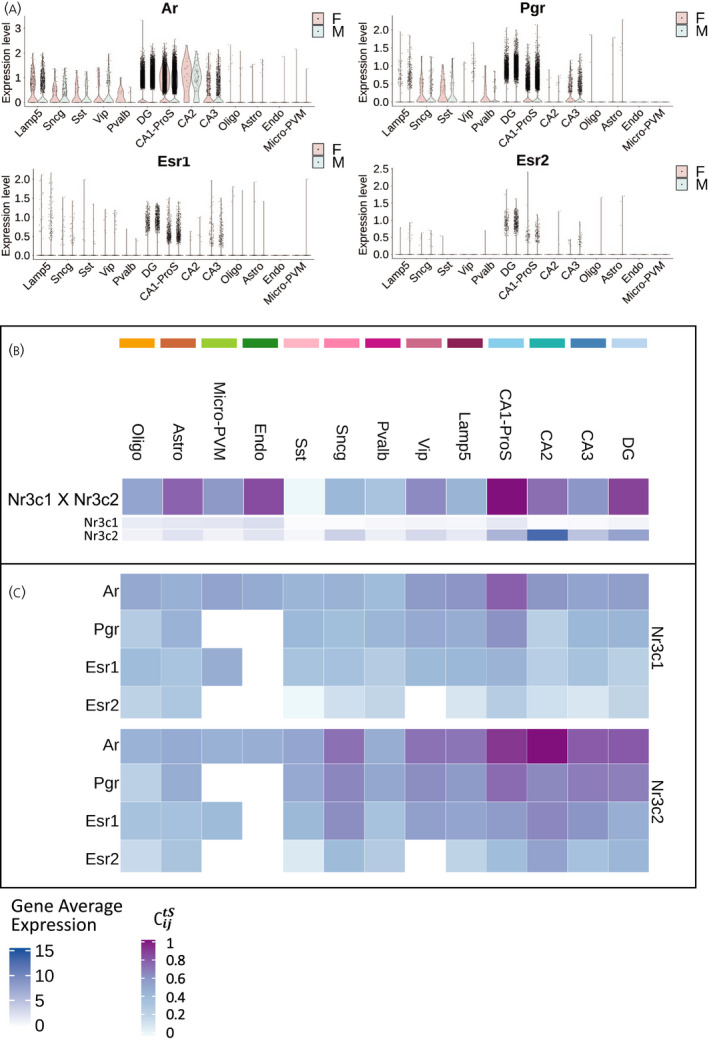
Cell type specificity of *Nr3c1* and *Nr3c2* co‐expression with sex hormone receptors. (A) Violin plots representing the log‐normalized expression (Expression level) of sex hormone receptors *Ar*, *Pgr*, *Esr1* and *Esr2* in cells obtained from female (F) and male (M) mice. (B) Heatmap representing the coupling score ${∁}_{\mathrm{ij}}^{\mathrm{tS}}$of *Nr3c1* with *Nr3c2*, and their respective log‐normalized average expression in mouse hippocampal cell types (*Gene Avg*. *Exp*). (C) Heatmap representing the coupling score ${∁}_{\mathrm{ij}}^{\mathrm{tS}}$of *Nr3c1* and *Nr3c2* with sex hormone receptors *Ar*, *Pgr*, *Esr1* and *Esr2* in mouse hippocampal cell types. Abbreviations: Nr3c1, nuclear receptor subfamily 3 group C member 1 (glucocorticoid receptor); Nr3c2, nuclear receptor subfamily 3 group C member 2 (mineralocorticoid receptor); Ar, androgen receptor; Pgr, progesterone receptor; Esr1 and Esr2, estrogen receptors *α* and *β*; F, female; M, male; Astro, astrocytes; Oligo, oligodendrocytes; Endo, endothelial cells; micro‐PVM, microglia/perivascular macrophages; Lamp5, lysosomal associated membrane protein family member 5; Vip, vasoactive intestinal peptide; Pvalb, parvalbumin; Sncg, synuclein gamma; Sst, somatostatin; DG, dentate gyrus; CA1‐ProS, cornus ammonis 1‐prosubiculum; CA2, cornus ammonis 2; CA3, cornus ammonis 3

*Ar*, *Pgr*, *Esr1* and *Esr2* were similarly expressed in cells that came from male or female mice in the scRNA‐seq with two subtle differences. Pvalb GABAergic neurons showed lower expression of *Ar* and *Pgr* in male cells, and CA3 had more positive cells and a slightly higher expression of *Pgr* in males. *Esr1* and *Esr2* were expressed in very few cells, with the highest expressing cell types being the DG granule cells and CA1‐ProS (Figure 4A).

We next determined cell type‐specific co‐expression between stress and sex hormone receptors. For this, we calculated a coupling score ${∁}_{\mathrm{ij}}^{\mathrm{tS}}$ based on basal average expression of pairs of genes in the different hippocampal cell types. Corticosteroid receptors (*Nr3c1* and *Nr3c2*) showed the highest coupling score in CA1‐ProS and were also highly co‐expressed in the DG, CA2, endothelial cells and astrocytes (Figure 4B; see also Table S3). The highest coupling score between stress and sex hormone receptors was found in neuronal cells. *Nr3c1* particularly co‐expressed with *Ar* and *Pgr* in CA1‐ProS, whereas *Nr3c2* not only co‐expressed with *Ar* mainly in glutamatergic, Lamp5, Vip and Sncg neurons, but also with *Pgr* in CA1‐ProS (Figure 4C; see also Table S3). The coupling scores between *Nr3c1* and *Nr3c2* and estrogen receptors were very low because of the absence of *Esr1* or *Esr2* expression in most cells. The highest coupling score for *Esr1* and *Nr3c2* was in CA2 and Sncg, certainly driven by the high *Nr3c2* expression.

We conclude that overall male and female mice have highly similar gene expression profiles for sex hormone receptors, and that co‐expression of sex‐ and stress hormone receptors is highly cell type specific.

### 3.5 *Nr3c1* and *Nr3c2* co‐expression with AF‐2 coregulators suggests cell type‐specific transcriptional modulation of GC signaling

**FIGURE 5:**
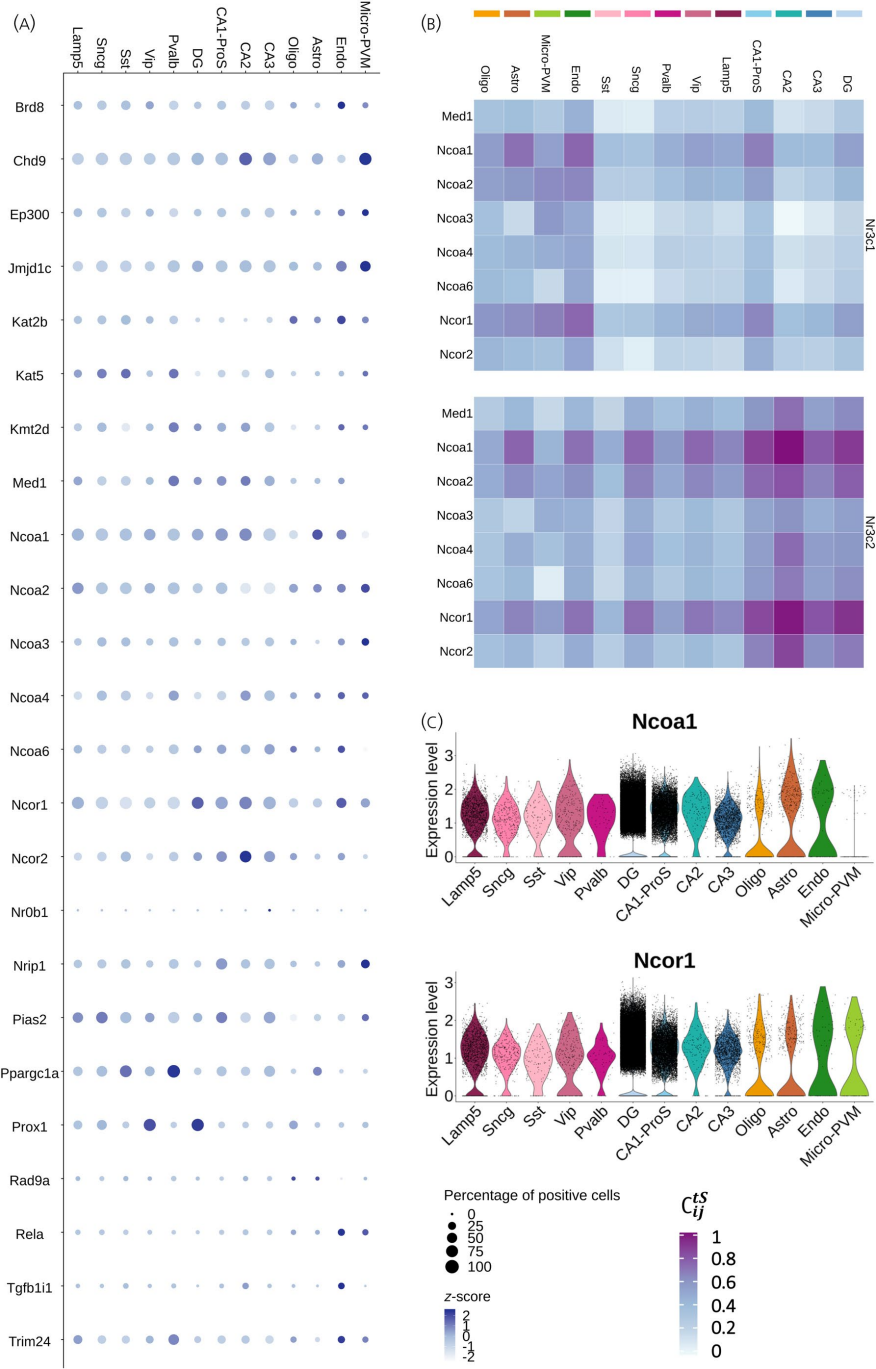
Cell type specificity of *Nr3c1* and *Nr3c2* co‐expression with nuclear receptor coregulators. (A) Dot plot representing both the centered log‐normalized average expression (*z*‐score) and the percentage of positive cells for 24 nuclear receptor AF‐2 coregulators known to interact with GR and/or MR according to an in vitro interaction screening assay.^24^ (B) Heatmap representing the coupling score ${∁}_{\mathrm{ij}}^{\mathrm{tS}}$of *Nr3c1* and *Nr3c2* with a subset of GR and MR coactivators and corepressors in mouse hippocampal cell types. (C) Violin plots representing the log‐normalized expression (Expression level) of the coactivator *Ncoa1* and the corepressor *Ncor1* in mouse hippocampal cell types. Abbreviations: Nr3c1, nuclear receptor subfamily 3 group C member 1 (glucocorticoid receptor); Nr3c2, nuclear receptor subfamily 3 group C member 2 (mineralocorticoid receptor); Astro, astrocytes; Oligo, oligodendrocytes; Endo, endothelial cells; micro‐PVM, microglia/perivascular macrophages; Lamp5, lysosomal associated membrane protein family member 5; Vip, vasoactive intestinal peptide; Pvalb, parvalbumin; Sncg, synuclein gamma; Sst, somatostatin; DG, dentate gyrus; CA1‐ProS, cornus ammonis 1‐prosubiculum; CA2, cornus ammonis 2; CA3, cornus ammonis 3; AF‐2, ligand‐dependent transactivation domain 2 (helix 12); *Med1*, mediator complex subunit 1; *Ncoa1*, nuclear receptor coactivator 1; *Ncoa2*, nuclear receptor coactivator 2; *Ncoa3*, nuclear receptor coactivator 3; *Ncoa4*, nuclear receptor coactivator 4; *Ncoa6*, nuclear receptor coactivator 6; *Ncor1*, nuclear receptor corepressor 1; *Ncor2*, nuclear receptor corepressor 2

Transcriptional coactivators and corepressors are key regulators of GC‐driven gene transcription. The presence of one particular coregulator can determine the outcome of GC signaling in a cell population.^35^, ^36^, ^37^, ^38^ In an *in vitro* screening assay, evidence was reported of 24 coregulators interacting with corticosteroid nuclear receptors: five with both receptor types, 17 with GR only and two with MR only.^24^ In scRNA‐seq data, each of these coregulators showed a specific expression pattern throughout different hippocampal cell types. For example, somewhat unexpectedly, *Ncoa2* was expressed in all cell types,^39^ and its highest expression level was found in microglia, whereas *Prox1* was mainly expressed in Vip GABAergic neurons and in the DG, where it was significantly enriched (log_2_FC = 1.47, *p* < .001) (Figure 5A; see also Table S1).

We further assessed co‐expression of AF‐2 coregulators with *Nr3c1* and *Nr3c2* (see Figure S2A and Table S3) for a subset of well‐characterized coactivators (*Med1* and *Ncoa* family) and corepressors (*Ncor1* and *Ncor2*) (Figure 5B). There was a clear co‐expression with the coregulators in non‐neuronal cells for *Nr3c1* and in glutamatergic neurons for *Nr3c2*. Interestingly, both *Nr3c1* and *Nr3c2* strongly co‐expressed with *Ncoa1* and *Ncor1*, which showed the exact same pattern of co‐expression throughout cell types. *Ncoa1* and *Ncor1* showed the highest coupling scores with *Nr3c1* and *Nr3c2* in CA1‐ProS, astrocytes and endothelial cells, and with *Nr3c2* in other glutamatergic neurons, as well as Vip and Sncg GABAergic neurons (Figure 5B). *Ncoa1* and *Ncor1* were expressed almost at the same level in all hippocampal cell types; except for microglia, which did not express *Ncoa1* (Figure 5C). Therefore, the co‐expression of these co‐regulators with stress hormone receptors is mainly driven by the cell specificity of *Nr3c1* and *Nr3c2* expression, with the notable exception of microglia.

### 3.6 Neurotransmitter and neuropeptide receptors differential co‐expression with *Nr3c1* and *Nr3c2* suggests synapse‐specific inputs

We next focused on neurotransmitter and neuropeptide pathways in the hippocampal glutamatergic tri‐synaptic path, which is the best characterized synaptic transmission route in the hippocampus. In this glutamatergic circuit, excitatory projections from the entorhinal cortex reach the DG granule cells through the perforant path, and the DG mossy fibers project to CA3 pyramidal neurons, which in turn stimulate CA1 neurons through the Schaffer collateral pathway.^40^ In addition to the tri‐synaptic path, CA1 also receive direct and strong excitatory projections from CA2.^41^ Although the sensory information mostly arrives in the DG, the CA‐regions also receive inputs from other brain regions. Afferent synapses to the tri‐synaptic path are not only glutamatergic, but also include neurotransmitters such as noradrenaline (NA), dopamine (DA) or serotonin (5‐hydroxytryptamine, 5‐HT), acetylcholine (ACh) and neuropeptides. We addressed the co‐expression of genes coding for NA, DA, 5‐HT, ACh and 33 neuropeptide receptors with *Nr3c1* and *Nr3c2* (Table S3), to determine how these pathways could interact with GC signaling in the hippocampal tri‐synaptic circuit.

**FIGURE 6:**
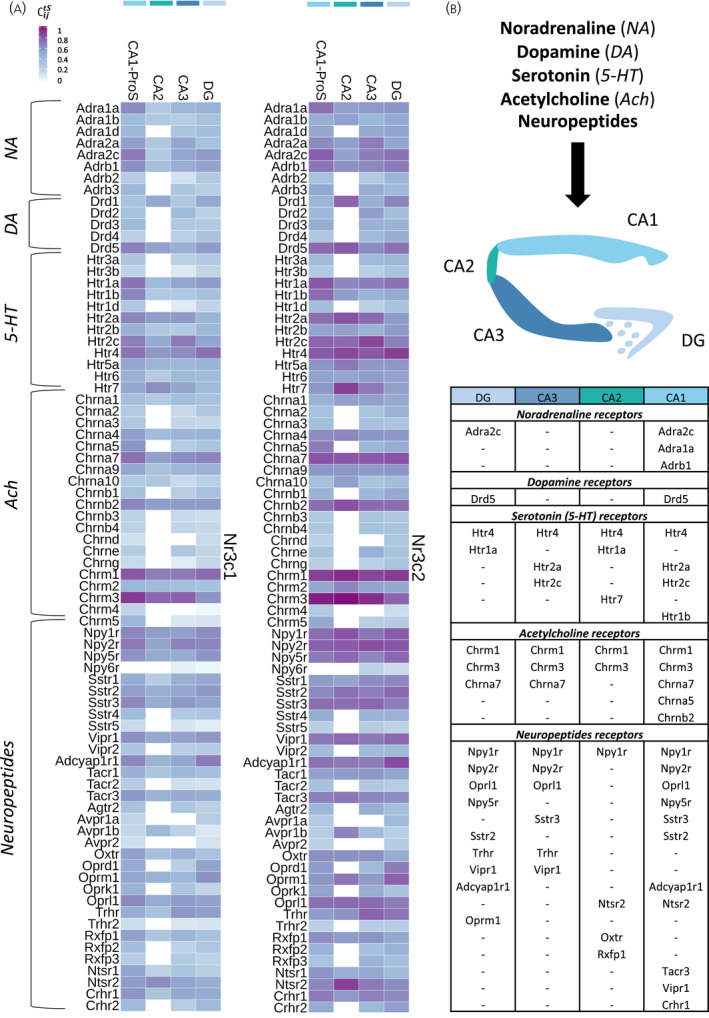
Cell type specificity of *Nr3c1* and *Nr3c2* co‐expression with neurotransmitter and neuropeptide receptors in the hippocampal tri‐synaptic pathway. (A) Heatmap representing the coupling score ${∁}_{\mathrm{ij}}^{\mathrm{tS}}$ of *Nr3c1* and *Nr3c2* with adrenergic; dopaminergic; serotoninergic; cholinergic and neuropeptides receptors in excitatory neurons of the hippocampal tri‐synaptic pathway. (B) Table of the neurotransmitter and neuropeptide receptors above threshold in terms of coupling with *Nr3c1* and *Nr3c2* expression (coupling score ${∁}_{\mathrm{ij}}^{\mathrm{tS}}$ > 0.6). Abbreviations: Nr3c1, nuclear receptor subfamily 3 group C member 1 (glucocorticoid receptor); Nr3c2, nuclear receptor subfamily 3 group C member 2 (mineralocorticoid receptor); DG, dentate gyrus; CA1‐ProS, cornus ammonis 1‐prosubiculum; CA2, cornus ammonis 2; CA3, cornus ammonis 3; NA, noradrenaline; DA, dopamine, 5‐HT, 5‐hydroxytryptamine; ACh, scetylcholine

NA receptors were mainly of the alpha‐1a, alpha‐2a/c and beta‐1 types. They co‐expressed with *Nr3c1* in CA1‐ProS, and also with *Nr3c2* in CA2, CA3 and the DG (Figure 6A, *NA*). For DA receptors, *Drd5* co‐expressed strongly with *Nr3c1* in CA1‐ProS and with *Nr3c2* co‐expressed in all glutamatergic neurons. *Drd1* co‐expressed with *Nr3c2* in CA2 and the DG (Figure 6A, *DA*). Many 5‐HT receptors were strongly co‐expressed with *Nr3c1* or *Nr3c2* in all regions of the tri‐synaptic circuit, particularly *Htr1a*, *Htr2a*, *Htr2c* and *Htr4* (Figure 6A, 5‐HT). The most consistent co‐expressed ACh receptors throughout the tri‐synaptic circuit were *Chrm1* and *Chrm3* (Figure 6A, *Ach*). Neuropeptide Y (NPY) receptors 1, 2 and 5 were strongly co‐expressed with *Nr3c2* in all cell types, whereas they were more specific to CA1‐ProS and the DG for *Nr3c1*, which reflects specificity of steroid receptors more than of these three types of NPY receptors. *Sstr2* and *Sstr3* were the most co‐expressed somatostatin receptors, whereas *Vipr1* was the most strongly co‐expressed vasoactive intestinal peptide receptor. *Adcyap1r1* (pituitary adenylate cyclase‐activating polypeptide type I receptor) was highly co‐expressed with *Nr3c1* in CA1‐ProS and with *Nr3c2* in all glutamatergic neurons. Tachykinin receptor Tacr3, opioid receptor Oprl1 and corticotropin‐releasing hormone (CRH) receptor Crhr2 were co‐expressed the strongest with *Nr3c1* in CA1‐ProS. *Nr3c2* co‐expressed with tachykinin, arginine‐vasopressin, oxytocin, opioid, thyrotropin‐releasing hormone, relaxin, neurotensin and CRH receptors in several glutamatergic synapses (Figure 6A, *Neuropeptides*).

We selected for the genes that had a coupling score above 0.6 both with *Nr3c1* or *Nr3c2* to obtain an overview of the strongest correlated neurotransmitter and neuropeptide receptors with GC signaling in the tri‐synaptic circuit (Figure 6B). For example, NA receptors are most robustly co‐expressed with *Nr3c1* and *Nr3c2* in the DG and CA1‐ProS.

Neurotransmitter and neuropeptide receptors co‐expression with corticosteroid receptors was more selective in GABAergic neurons and non‐neuronal cells. For example, in microglia, *Nr3c1* (and *Nr3c2*) showed high co‐expression with *Adrb1* and *Adrb2*. The coupling score with *Ntsr2* was particularly high in astrocytes (see Figure S2B).

### 3.7 *Nr3c1* and *Nr3c2* escape *de novo* GRN analysis

**FIGURE 7:**
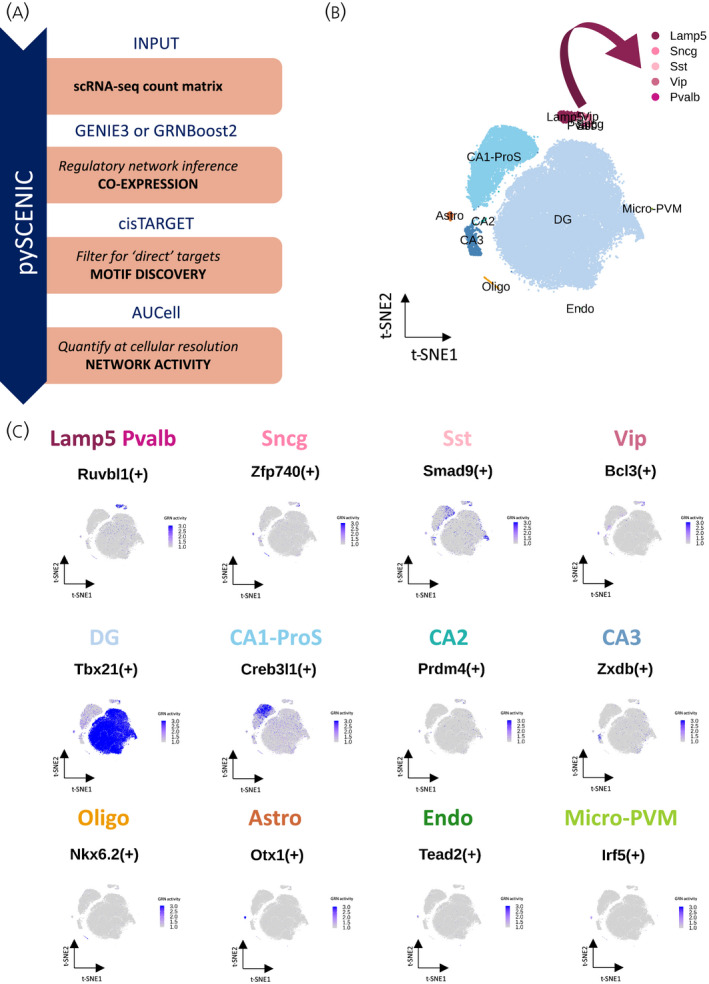
Mouse adult hippocampus gene regulatory networks (GRNs). (A) Description of the pySCENIC pipeline. (B) Dimensional reduction (t‐SNE) representation of mouse hippocampal cells grouped based on GRN activity similarities and assigned to known cell types. (C) t‐SNE representation of each hippocampal cell population most active GRN activity level per cell; scaled from 1 to 3. The sign (+) allows the distinction between a transcription factor gene (e.g., *Neurod2*) and this same transcription factor network; e.g Neurod2(+). Abbreviations: scRNA‐seq, single‐cell RNA sequencing; GRN, gene regulatory network; Astro, astrocytes; Oligo, oligodendrocytes; Endo, endothelial cells, micro‐PVM, microglia/perivascular macrophages; Lamp5, lysosomal associated membrane protein family member 5; Vip, vasoactive intestinal peptide; Pvalb, parvalbumin; Sncg, synuclein gamma; Sst, somatostatin; DG, dentate gyrus; CA1‐ProS, cornus ammonis 1‐prosubiculum; CA2, cornus ammonis 2; CA3, cornus ammonis 3

It is known that cell‐specific gene regulation relies essentially on coordination of the activity of TFs.^42^ Recent progress in high‐throughput sequencing allows the reconstruction of TF downstream networks. We applied the pySCENIC pipeline to determine whether we could identify putative MR and GR dependent regulatory networks in particular cell types.^15^ The pySCENIC workflow is divided into three steps: first, it computes co‐expression modules of a TF with all correlated genes based on the scRNA‐seq count matrix. Then, these co‐expression modules are further refined by selecting genes with the TF‐specific DNA motif in their promoter region, generating the GRN modules. Finally, the refined GRN activity is measured in each individual cell, by scoring the component gene expression per GRN, and is used for new clustering (Figure 7A).

In this analysis, we based the t‐SNE dimensional reduction on GRN activity, rather than gene expression. The t‐SNE included the same 13 cell types, but the clustering grouped the cells differently. The most notable change was the disappearance of GABAergic neurons specificities. These neurons grouped together as one cluster, which means that all GABAergic neuronal types have very similar GRN activity profile (Figure 7B), as described previously using pySCENIC in scRNA‐seq brain data.^15^, ^43^ During the refinement of co‐expression modules into GRNs, the co‐expression modules with less than 80% of genes containing a binding site for the TF in their promoter region were excluded. *Nr3c1* and *Nr3c2* GRN activity could not be calculated as a result of not passing this threshold of motif discovery. Nevertheless, the GRN analysis allowed the identification of some cell type‐specific gene networks in the mouse hippocampus (see Figure S3A and Table S4). For example, the neuronal GRN *Hsf3*(+), the GABAergic GRN *Maf*(+) and the glutamatergic GRN *Neurod2*(+) showed cell type specific activity (Figure S3B). To further characterize the mouse hippocampus cell diversity, we performed a differential activity analysis on GRNs to identify the most active GRN for each cell type (Figure 7C; see also Table S5). GRNs were more specific in non‐neuronal cells. For example, *Otx1*(+) is the most active GRN in astrocytes, being expressed in 94% of astrocytes and only 1% of all‐other cells, with an activity enrichment log‐fold change of 4.24 (see Table S5).

Although we could not determine genes involved in *Nr3c1*(+) and *Nr3c2*(+) regulatory networks and their differential activity in hippocampal cell types, the pySCENIC allowed for a better characterization of other TF downstream networks in mouse hippocampus. This can in turn be important in determining the cellular context of stress hormone receptor activity.

## 4 DISCUSSION

We set out to describe the cell‐specific gene expression in the hippocampus aiming to better understand MR and GR‐mediated signaling. In a non‐treated context, corticosteroid receptor genes *Nr3c1* (GR) and *Nr3c2* (MR), classic GC responsive genes and newly categorized target genes showed a very heterogenous basal expression throughout hippocampal cell types, and likely predicted cell type‐specific responsiveness to GC signaling activation. Furthermore, the results on co‐expression suggested cell type‐specific crosstalk between sex and stress hormones, as well as a possible cell type‐specific transcriptional coregulation. Our results also summarize the heterogeneity in stress hormone receptor co‐expression with neurotransmitter and neuropeptide receptors in the tri‐synaptic hippocampal circuit. Finally, despite providing no further insight on GR and MR downstream GRN cell specificity, the pySCENIC pipeline revealed the cell‐specific activity of 376 TF GRNs in the mouse hippocampus. These later results further emphasize the hippocampal cell heterogeneity in terms of gene transcription activity.

Our results confirm high MR mRNA expression in glutamatergic neurons (Figure 2D), in line with its previously reported presence, and its role in mediating effects in hippocampal pyramidal and granule cell excitability.^44^, ^45^, ^46^, ^47^, ^48^ MR expression in CA2 glutamatergic cells stands out, and a recent study showed that neuronal MR deletion resulted in the disappearance of CA2 molecular identity.^49^ It is interesting to note that GABAergic neurons have appreciable levels of MR mRNA. To date, based on predominant presence in the granular and pyramidal cell layers, the glutamatergic cells have received most attention. However, the widespread presence of MR challenges the notion of purely cell‐autonomous effects in glutamatergic neurons. This expands the focus of future work looking into the basis of the MR‐mediated effects on cognitive and emotional processing.^50^, ^51^ On the other hand, MR binding to DNA earlier was linked to NeuroD factors, and this appears to reflect mainly glutamatergic neurons (see Figure S1B,C). Immunohistochemical co‐expression studies will therefore be a valuable addition to this, as well as other findings at the mRNA level.

Our data for GR also validate some known notions, such as the relatively low expression of GR mRNA in CA3 pyramidal cells (Figure 2D).^52^, ^53^ The presence of both receptor types in the glutamatergic CA1 neurons fits well with GR and MR cell‐autonomous opposite effects in CA1.^54^ GR is certainly expressed in DG granule cells, although the percentage of positive cells is, perhaps surprisingly, modest. This may explain why corticosterone‐sensitivity of DG excitability and gene expression is markedly different from CA1 pyramidal neurons.^55^, ^56^, ^57^ The DG is arguably the most complex structure in the hippocampus in terms of cellular diversity and organization.^58^ A possible reason for the DG heterogeneity is hippocampal neurogenesis, leading to cells in different stages of neuronal maturation. Recent results suggest that neuronal progenitor cells and their progeny have intrinsic GC sensitivity and display a dorsoventral differential response to long‐term GC exposure.^59^ These results could explain the contrast that we observed in MR expression. The data supported differential GC sensitivity in the DG but did not allow further subdivision in DG cells because of their overall very similar pattern of gene expression. The level of clustering that we used in the deeper analysis of the DG divided the region in only six subclusters. It is likely that more depth in the scRNA‐seq associated with clustering based on neurogenesis markers would provide further insights on MR expression in neurons at different maturation stages.

GR mRNA expression was also high in oligodendrocytes, astrocytes, microglia and endothelial cells (Figure 2D). Functionality of GR in glial cell types has previously been established, for example with cell type‐specific knockout mouse models.^60^, ^61^, ^62^ Indeed, in a mouse model for Cushing’s disease (AdKO), we observed clear changes for astrocytes, microglia and oligodendrocytes.^63^ For all of these cell types, effects of GCs, stress and/or GR antagonists (direct and indirect) have been reported in rodents and human studies.^64^, ^65^, ^66^, ^67^ Specifically, microglial cells are clearly responsive to stress and GCs, and have recently been reported to play a role in synaptic plasticity.^68^, ^69^ Interestingly, the signaling repertoire of GR in microglia is unique for the brain, in that *Ncoa1* (coding for the steroid receptor coactivator‐1 or SRC‐1) is hardly expressed, and *Ncoa2* (coding for the SRC‐2/GRIP1) may be a predominant GR coregulator (Figure 5A), analogous to immune‐modulatory GR effects in the periphery.^70^, ^71^ A cell type‐specific coregulator repertoire may allow more selective targeting of GR using selective receptor modulators that distinguish between downstream signaling pathways.^35^, ^36^, ^37^, ^38^ For example, in an epilepsy model, treatment with the selective GR modulator CORT108297 limited reactive microgliosis in the mouse DG without affecting an increase in astrogliosis.^72^

The set of MR/GR target genes used in the present study relied on previous studies that all addressed brain or neuronal tissue. Yet, there were many differences in species, genetic background and age, exact tissue, type of intervention, dosage and type of GC used, and latency between treatment and sample collection (see Table S2). We could not provide a complete description of the conditions across the studies because they sometimes failed to mention housing and light cycle conditions, the animal sex or the timing of their intervention. Therefore, although we trust our criteria selected robustly responding GC target genes, the list is by no means exhaustive. Expression of MR/GR target genes clearly differed between cell types, but basal expression does not necessarily reflect the cell type‐specific GC responsiveness. For example, *Sgk1* is known to be strongly and apparently quite selectively induced in white matter.^73^, ^74^ However, our results showed that *Sgk1* basal mRNA levels are high in all neuronal cell types, oligodendrocytes and microglia (Figure 3A). This is an example of a gene where basal expression does not fully correlate with MR and/or GR mediated effects. However, only very few target genes show such almost binary on–off responses after GC elevations. Therefore, we expect that increased levels of *Fkbp5* mRNA reflect responses in glutamatergic neurons, and those of *Tsc22d3* mRNA mainly responses in other cell types. An additional argument in favor of basal expression predicting “target‐ness” is that an increased mRNA level in a relatively small cell population will be diluted by steadily high expression levels in other more abundant cell types. However, this all remains to be confirmed based on experimental data addressing responses in specific cell types. The uncertainty of cell‐specific target genes applies to a lesser extent for genes that are downregulated because this can only occur in cell types that initially expressed the gene of interest. Specific expression of downregulated genes appears to concern mainly non‐neuronal cell types (Figure 3C; see Figure S1D), for microglia clearly pointing to GR rather than MR‐mediated responses.

Susceptibility and prevalence of stress‐related neuropsychiatric and neurodegenerative pathologies differ between men and women^75^, and the prevalence of these stress‐related disorders increases in females upon drastic hormonal changes.^76^ Many of these disorders have been associated with altered structure, function and neurogenic processes within the hippocampus,^77^, ^78^, ^79^, ^80^, ^81^ suggesting a possible sex dimorphism in GC effects on hippocampal function. Our results showed that cell‐specific GR and MR mRNA levels correlated substantially with AR and PR mRNA (Figure 4C). This could suggest a direct crosstalk between those receptors because AR and PR can bind to GREs.^82^ On the other hand, interactions with ER likely do not have a great impact in the hippocampus, given the low expression of *Esr1 and Esr2* (Figure 4A and C). Thus, the quite large literature on estrogen effects on hippocampal function^83^, ^84^, ^85^ points to involvement of membrane estrogen receptors^86^, ^87^ and/or interactions in afferent brain areas.

The hippocampal tri‐synaptic path receives various inputs from other brain regions and harbors a large diversity of synapses with receptors for NA, DA, 5‐HT, ACh and neuropeptides. In our results, CA1 showed the highest number of NA, DA, 5‐HT and ACh receptors that were strongly co‐expressed with GR and MR (Figure 6B). Previous studies showed that NA, DA and 5‐HT can suppress the perforant path input to CA1 by reducing postsynaptic potentials.^88^ This suggests a possible interaction between GR/MR and neurotransmitter receptor signaling that could influence CA1 synaptic activity, conforming with the early work by Joëls et al.^100^ Basal forebrain cholinergic neurons that project to the hippocampus are involved in stress adaptation and cognition.^89^ The cholinergic system interacts with GC signaling in processes such as hippocampal‐dependent memory reconsolidation.^90^ Our results suggest that the ACh receptors likely to be involved in this crosstalk are *Chrm1*, *Chrm3* and *Chrna7* (Figure 6B). In humans, higher NPY levels in serum and plasma were correlated with adaptive coping following stress as well as PTSD resilience.^92^, ^93^, ^94^ A study in rats suggested that NPY interneuron activation in the DG contributed to trauma resilience in a model for PTSD.^94^ Our results suggest that *Npy1r*, *Npy2r* and *Npy5r* expression is highly coupled with GR and MR mRNA levels in the DG (Figure 6B). Conceivably, NPY and GC signaling communicate via interaction of those receptors in the rodent DG (inter)neurons. Hippocampal oxytocin was found to be important for social discrimination,^95^ and oxytocin can prevent stress‐induced hippocampal synaptic dysfunction and impairment of long‐term potentiation and memory.^96^ Our results suggest that oxytocin signaling interference with GC signaling is mainly restrained to the hippocampal cornu ammonis region (Figure 6B). Our data also confirm the predominant role of CA2 specific AVPR1B receptors in stress‐related signaling, in conjunction with MR (Figure 6A).^49^, ^98^

Glucocorticoid receptor and MR activation may affect neuronal development,^99^ as exemplified in CA2 pyramidal cells for MR^49^ and the DG granule cells for GR.^100^ This may be linked to corresponding downstream regulatory pathways. However, when looking for transcriptional networks, GR and MR did not meet the selective criteria for the pipeline motif discovery because their co‐expression modules had <80% of genes with a detected binding site in their promoter region. The pySCENIC motif discovery is limited to 10 kb down‐ and upstream of gene transcription start sites, whereas GR (and supposedly MR) binding sites are often further from their target gene start sites.^21^ For hippocampal target genes, an *in silico* GRE interspecies screening of GC‐responsive genes showed that GREs were between 30 kb downstream and 175 kb upstream of transcription start sites of GR target gene start site, with a typical example being *Adra1b* that is co‐expressed with GR in pyramidal cells (Figure 6A).^23^ In addition, the inability for the pySCENIC pipeline to detect MR network may have been related to an overestimation of potential MR target genes. MR mRNA levels were high in most cells in the hippocampus and significantly correlated with a total of 7319 genes. Consequently, its direct genomic targets may have been diluted by other correlated genes, leading to loss of statistical power. Nevertheless, the dominant co‐expression modules provided the cellular context in which MR and GR can bind to chromatin, and this may well be relevant, as exemplified by the Neurod2(+) GRN that may be linked to MR target genes (see Figure S3B).

Although our data in part recapitulate previous published transcriptomic studies, the cell type‐specific expression of genes that potentially interact with MR and GR allows for a reinterpretation of GC signaling in the adult mouse hippocampus. With the lack of an actual single cell transcriptomic dataset after GC treatment, the cell type‐specific expression of MR/GR downstream targets suggests gene markers to study the responsiveness of particular cell types. Moreover, the co‐expression of potentially interacting factors, such as other steroid receptors and transcriptional coregulators, defines where direct interactions can take place, and may help to more specifically target the receptors with selective modulators.^38^ We hope that the results will allow the formulation of more defined future hypotheses on stress hormone effects on hippocampal function.

## CONFLICT OF INTEREST

The authors declare that they have no conflicts of interest.

## AUTHOR CONTRIBUTIONS

**Eva M. G. Viho:** Conceptualization; data curation; formal analysis; investigation; methodology; project administration; visualization; writing – original draft. **Jacobus C. Buurstede:** Data curation; formal analysis; writing – review and editing. **Jari B. Berkhout:** Data curation; formal analysis; writing – review and editing. **Ahmed Mahfouz:** Conceptualization; investigation; methodology; supervision; writing – review and editing. **Onno C. Meijer:** Conceptualization; funding acquisition; investigation; project administration; supervision; writing – review and editing.

### PEER REVIEW

The peer review history for this article is available at https://publons.com/publon/10.1111/jne.13072.

### OPEN RESEARCH BADGES

This article has been awarded Open Materials, Open Data Badges. All materials and data are publicly accessible via the Open Science Framework at https://github.com/eviho/10XHip2021_VihoEMG.git.

## Supporting information

Fig S1

Fig S2

Fig S3

Table S1

Table S2

Table S3

Table S4

Table S5

## Data Availability

The bulk RNA‐seq data have been deposited in NCBI’s Gene Expression Omnibus and are accessible through GEO Series accession number GSE184924. The code that supports the findings of this study is openly available in the GitHub repository (https://github.com/eviho/10XHip2021_VihoEMG). The datasets used in the code can be downloaded from Zenodo (https://doi.org/10.5281/zenodo.5729701).
