# Supplementary figures and images for: Cell type specificity of glucocorticoid signaling in the adult mouse hippocampus
Source: J Neuroendocrinol. 2021 Dec 22;34(2):e13072. doi: 10.1111/jne.13072 (PMC9286676; doi:10.1111/jne.13072)

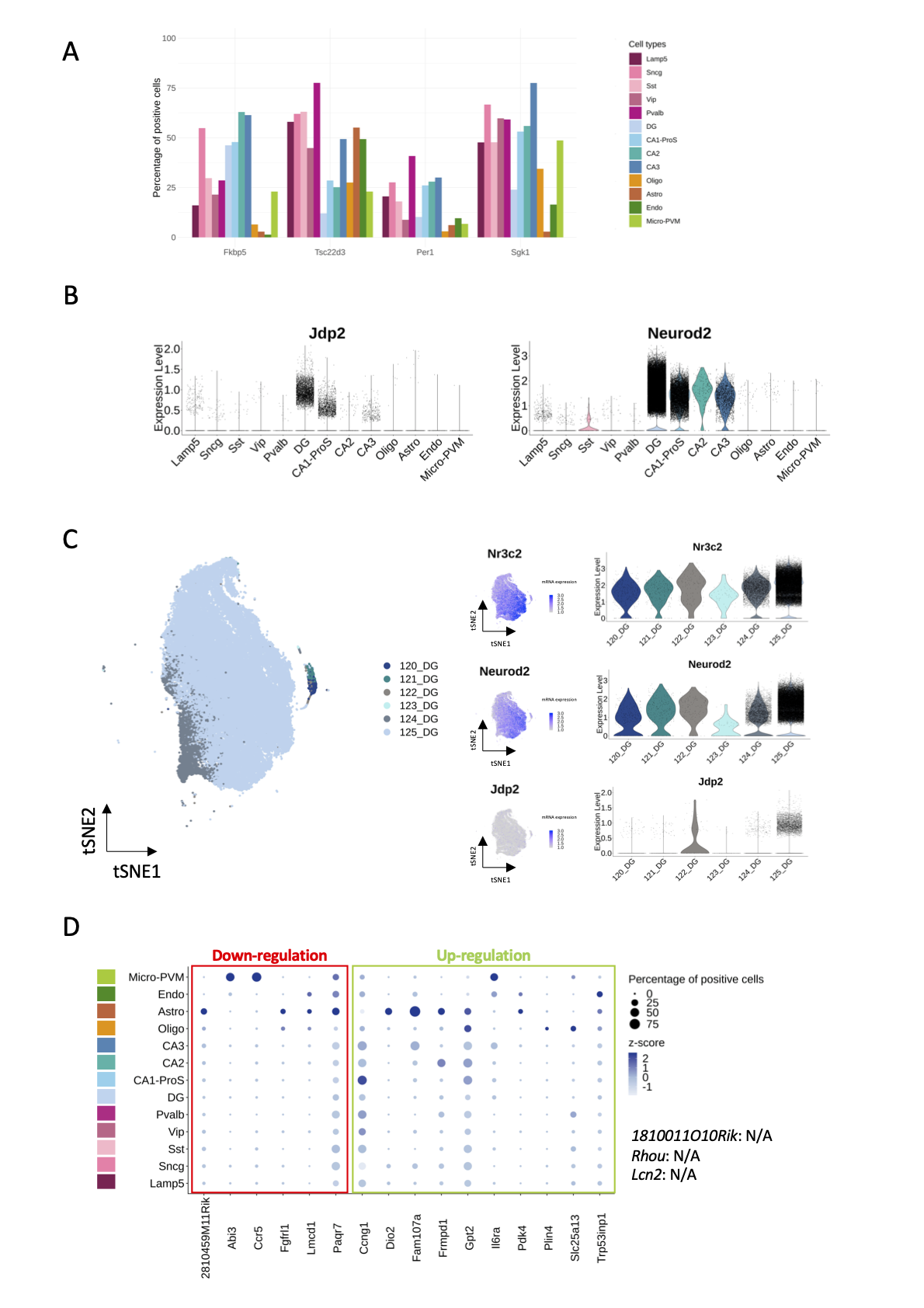

Supplement: Supplementary file 1 — Fig S1 [file JNE-34-0-s004.tiff]

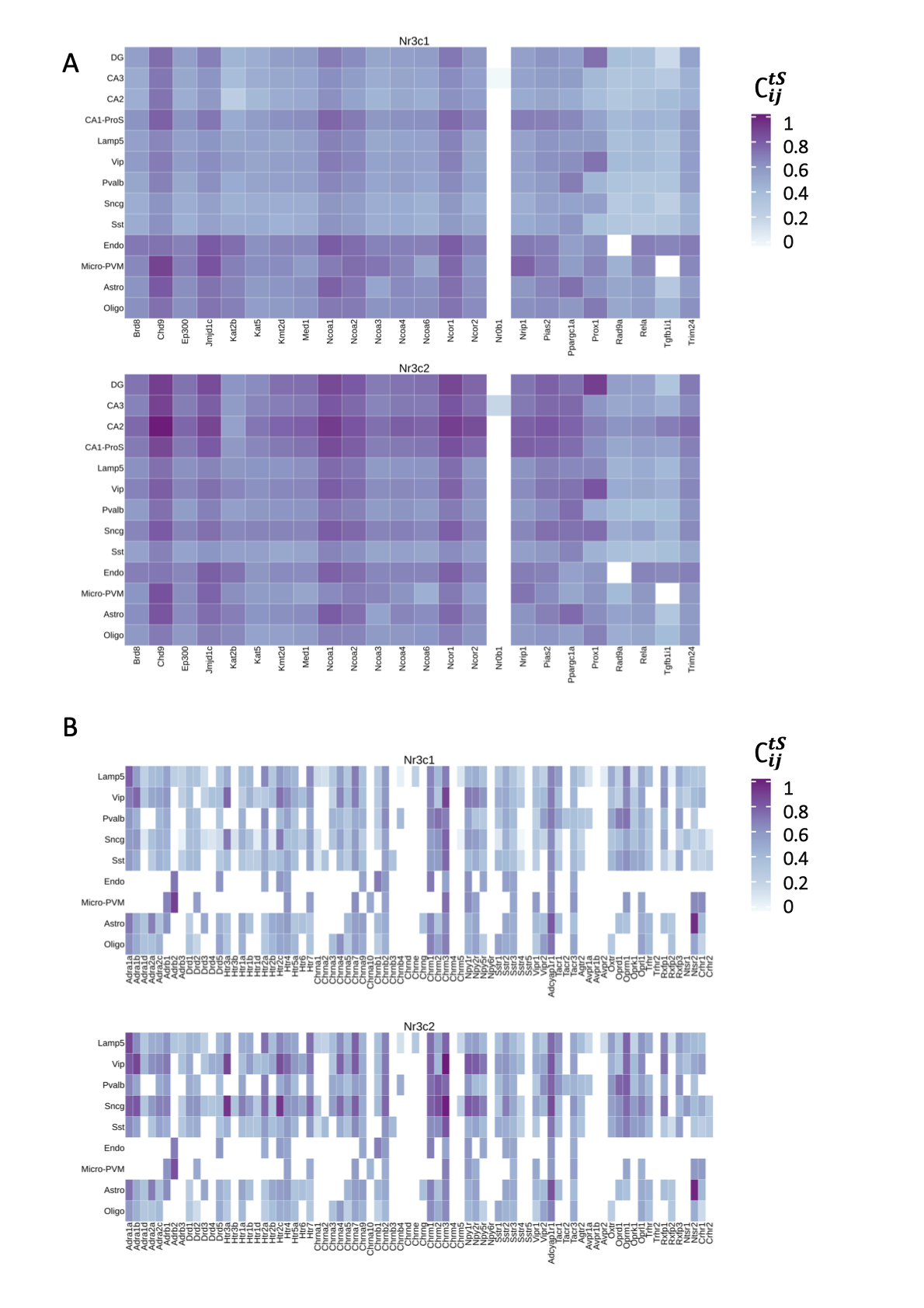

Supplement: Supplementary file 2 — Fig S2 [file JNE-34-0-s003.tiff]

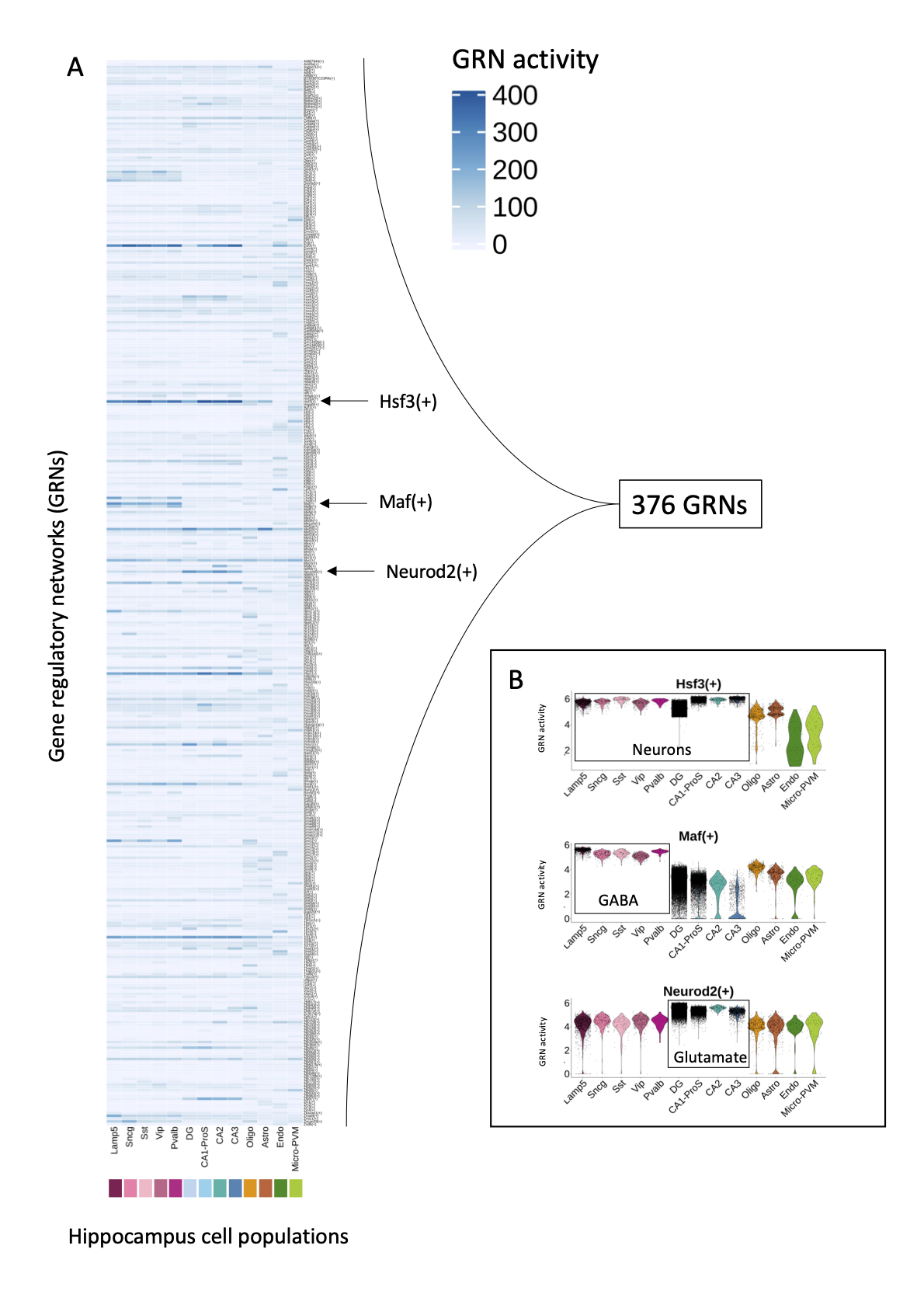

Supplement: Supplementary file 3 — Fig S3 [file JNE-34-0-s007.tiff]
